# Four-Year Monitoring Survey of Pesticide Residues in Tomato Samples: Human Health and Environmental Risk Assessment

**DOI:** 10.3390/jox15050171

**Published:** 2025-10-20

**Authors:** Alessandro Atzei, Hamza Bouakline, Francesco Corrias, Alberto Angioni

**Affiliations:** 1 Department of Life and Environmental Sciences, University of Cagliari, Monserrato, 09042 Cagliari, Italy; francesco.corrias@unica.it; 2 Environment and Applied Chemistry Laboratory (LCAE), Faculty of Sciences, Mohammed First University, Oujda 60000, Morocco; h.bouakline@ump.ac.ma

**Keywords:** monitoring survey, pesticide residues, multiresidues methods, risk assessment, consumer health

## Abstract

A four-year survey was conducted to monitor the presence of multiple pesticide residues contaminating tomatoes, with the aim of evaluating the potential health and environmental risks. A multiresidue liquid chromatography–triple mass spectrometry with a multiple reaction monitoring (LC-MS/MS-MRM) method was fully validated and used to test 252 pesticides in 360 samples analysed. According to SANTE guidelines, the proposed method was considered suitable for the purpose. Dietary risk assessment was conducted using the Hazard Quotient (HQ) approach and the European Food Safety Authority (EFSA) Pesticide Residue Intake Model; meanwhile, the cumulative environmental risk assessment was conducted using the Concentration Addition (CA) and Independent Action (IA) methods. Data obtained revealed multiple contaminations in most fields examined over the years. Twenty-two pesticide residues were identified, comprising 68.2% fungicides, 27.3% insecticides, and the remaining 4.5% acaricides. Higher levels were detected for Boscalid in 2022 in three fields, with an average value of 0.42 mg/kg. Multi-residue contamination occurred each year; the lowest abundance was detected in 2023 (3.9%), and the highest in 2022 (12.3%), with 5 pesticide residues as the maximum number of compounds detected in one sample in 2022. The consumer risk assessment identified no potential health concerns for adults or toddlers, and the combined risk was considered acceptable. The environmental assessment showed maximum cumulative ratio (MCR) values that were always ≥1, indicating a contribution to the toxicity of the mixture, only slightly higher than that of the single compound with the highest toxicity. The results of this study highlight the critical need to include cumulative dietary exposure assessments in pesticide risk evaluations, especially for food products that are susceptible to contamination by multiple residues.

## 1. Introduction

The agricultural food production system depends on the use of plant protection products (PPPs) to preserve crops from pests and decay worldwide. The use of PPPs has an impact on human health and the environment [1]. Even when pesticide treatments are carried out following good agricultural practices, there is a possibility that these compounds may leave quantifiable residues on the commodities. Moreover, multiple residues could be encountered when multiple pesticides are applied, and the toxicological synergistic or additive effect cannot be predicted [2]. The European Green Deal with the farm-to-fork strategy highlights the necessity to decrease and progressively eliminate the use of pesticides. The European Union’s (EU) key actions are represented by the zero-pollution action plan and the biodiversity strategy, as well as the regulation on the sustainable use of pesticides [3]. These measures implement integrated pest management (IPM) methods, using products with lower impact on human health and the environment, prioritising non-chemical alternatives and setting up appropriately sized buffer zones [4]. Regulation (EC) No. 396/2005 and its amendments establish maximum residue levels (MRLs) for food products and animal feed. MRLs represent the legal limit and are obtained by correlating a complete dataset on toxicology and residue behaviour, together with methods of analysis [5]. However, zero-residue commodities reflect the current practices of food producers, indicating levels of pesticide residue below the limit of quantitative determination (LOQ) of official analytical methods [6].

With this perspective, the analysis should search for authorised and unauthorised pesticides for a specific crop. However, pesticide applications in crops often involve a mixture of PPPs, leading to complex pesticide mixtures; therefore, multiresidue methods capable of analysing a considerable number of pesticides in one spot represent the current challenge for analytical laboratories and food factories in monitoring purposes. These methods are mainly based on LC-MS/MS and GC-MS/MS (gas chromatography/triple mass spectrometry) techniques [7]. EU monitoring programmes are set annually according to Implementing Regulation (EU) 2024/989 and comprise 10 foodstuffs of plant origin and 208 parameters, 2 of animal origin and 33 parameters. In 2026, the foodstuffs selected will be oranges, pears, kiwis, cauliflower, onions, carrots, potatoes, beans (dried), rye (grains), brown rice (=de-husked rice), poultry fat, and beef liver [8].

Among the most cultivated vegetables in the EU, tomatoes (*Solanum lycopersicum* L.) account for almost 20%, with nearly a quarter used for processing (juices, canning, purees, sauces, and ketchup) [9].

Italy accounts for 15% of world production and 56% of the European crop; 60% of Italian processed tomatoes are exported worldwide. In Sardinia, 500 hectares are cultivated on average, producing nearly 500,000 quintals of industrial tomatoes, with 60% grown in the Medio Campidano and 40% in the Oristano area (data not published, supplied by the processing industry).

Tomatoes’ primary pathogens include fungal infections such as late blight (*Phytophthora infestans*) and *Alternaria* spp., as well as bacterial diseases and insects such as *Tuta absoluta*, *Helicoverpa armigera*, *Trialeurodes vaporarium*, and *Agrotis ipsilon* [10]. In Italy, the sustainable use of pesticides is regulated by the National Action Plan, allowing the use of 159 pesticides on tomatoes. The authorised compounds consist of fungicides (68), insecticides (54), nematicides (13), acaricides (12), herbicides (16), plant growth regulators (4), molluscicides (2), and pheromones (2) [11,12]. In 2020, the Italian Ministry of Health published a report on the control of residues of plant protection products in food, highlighting that out of a total of 4759 samples, half of the samples (54.6%) were found to be free from detectable residues, and 43.9% reported residue values below the legal limit, while the remaining 1.5% reported residues above the legal limit [13]. Ouakhssase et al. monitored 432 pesticides in tomato samples from a local market in Morocco. The results showed 56% of samples were contaminated with one or more pesticide residues [14]. El Sherif et al. reported the recovery of 19 pesticides on 18 tomato samples from an Egyptian local market, mostly with multiresidue contamination [15]. Monitoring plans are usually carried out on samples collected in the market or during imports from foreign countries and are intended to verify compliance with the legal limits for authorised pesticides, not considering a risk assessment for cumulative contamination. These plans provide information on the contamination of ready-to-eat plant products. However, data on products intended for industrial processing are accessible only to manufacturing companies. Therefore, there is a lack of cumulative risk assessment studies focusing on pre-processing industrial commodities both from a specific region and over multiple consecutive years.

This work presents the results of a four-year monitoring survey conducted in Sardinia, which aimed to evaluate the contamination of tomatoes with pesticide residues after field treatment before harvest for processing into by-products for industrial use. Analyses of pesticide residues were performed by UHPLC-MS/MS. The paper reports on the evaluation of pesticide multiple sample contamination risk assessment vs. human dietary and the environmental compart.

## 2. Materials and Methods

### 2.1. Standards and Reagents

Acetonitrile (ACN) and methanol were LC/MS-grade solvents purchased from Sigma Aldrich (Milan, Italy). Ammonium formate 5 M (product code: G1946-85021, Agilent, Milan, Italy), formic acid (reagent-grade purity > 95%) and certified analytical standards (98.0% purity, Table S1) of pesticides at 100 mg/L in ACN (LC/MS Pesticide Comprehensive Test Mix Kit, part number: 5190-0551) were from Agilent Technologies (Milan, Italy). An intermediate solution of the pesticide mixture was prepared at 1 mg/L in ACN. Calibration curves consisting of five diluting points were prepared daily from the intermediate solution in tomato extract, free of pesticides. QuEChERS salt1 (part number: 5982-6650, Agilent, Milan, Italy) and salt2 (part number: 5982-5056, Agilent, Milan, Italy) were used for extraction and purification. A MilliQ Millipore purification system produced water with a conductivity below 18.2 MΩ (MilliQ Integral, Merck, Milan, Italy).

### 2.2. Sample Collection and Processing

Tomatoes at industrial maturity were collected according to the Commission Directive 2002/63/EC [16] over four consecutive years from 2021 to 2024, from all cultivated fields in the provinces of Medio Campidano and Oristano for a total of 360 samples (126-2021, 81-2022, 76-2023, and 79-2024) and 1375.4 ha sampled. After harvest, tomato samples were brought to the laboratory and stored in a refrigerator at 4 °C until processing (within 24 h after collection). The entire sample collected (5 kg) was chopped and homogenised using a semi-industrial blender; therefore, an aliquot was withdrawn and subjected to the extraction procedure. Three replicates for each sample were performed.

### 2.3. Extraction Procedure

Tomato samples were extracted according to Corrias et al. [17]. Briefly, ten grams of the homogenised sample were weighed into a 50 mL tube, mixed with 10 mL of ACN, and vortexed (Reax Top, Heidolph, Schwabach, Germany) for 1 min. Subsequently, 6.5 g of QuEChERS salt1 was added, and the tube was vortexed for 2 min and agitated in a rotary shaker for 15 min. The sample was centrifuged for 5 min at 1.957 relative centrifugal force (RCF) and 10 °C (Centrifuge 5810 R, Eppendorf AG, Hamburg, Germany). The supernatant (5 mL) was collected and transferred into a 15 mL tube containing 1 g of QuEChERS salt2. The tube was vortexed for 2 min, followed by agitation on a rotary shaker for 15 min. The solution was centrifuged for 5 min at 1.957 relative centrifugal force (RCF) and 10 °C. The organic fraction was filtered through a 0.45 μm PTFE filter (Thermo Scientific, Milan, Italy) and transferred into a 1.8 mL vial for UHPLC-MS/MS analysis immediately after the preparation step.

### 2.4. UHPLC-MS/MS Analysis and Method Validation

The analytical method was validated following the SANTE/11312/2021 guidelines [18]. The multiresidue UHPLC-MS/MS-MRM method updates a previously validated method by adding 136 pesticides for a final multiresidue analysis of 252 compounds [17]. Briefly, a Triple Quad 6470-1290 Infinity LC-MS/MS system (Agilent Technologies, Milan, Italy) was used with a ZORBAX Eclipse Plus C18 (2.1 × 150 mm, 1–8 μm) column and a binary gradient, H_2_O 5 mM in ammonium formate + 0.1% formic acid (A), and methanol 5 mM in ammonium formate + 0.1% formic acid (B). The analytical parameters evaluated included linearity, matrix effect, mean recovery (RE%), repeatability intra-day precision (RSDr), intermediate precision inter-day precision (RSDWR), and limit of quantification (LOQs). Pesticide-free tomato samples from organic growing fields were used as a blank matrix for calibration standards, recoveries, and matrix effect. Method linearity was assessed based on the coefficient of determination (r^2^) of five-point calibration curves constructed for each compound with concentrations ranging from 0.001 to 0.1 mg/L.

Expanded measurement uncertainty (U, with a coverage factor of k = 2, used to obtain a confidence interval of 95%) (Equation (1)) was calculated within the lab validation approach by combining component uncertainties of reproducibility and trueness (u_c_, with a coverage factor of k = 2 to achieve a confidence level of approximately 95%) (Equation (2)) [19]. Expanded uncertainty was calculated according to the following equations for all compounds:

U = 2 × u_c_ (1)

u_c_ = √(u(Rw)^2^ + u(bias)^2^) (2)

U = expanded measurement uncertainty; u_c_ combined measurement uncertainty.

u(bias) = uncertainty component for the bias (trueness).

u(Rw) = uncertainty component for the precision (within lab reproducibility of the recovery).

### 2.5. Risk Assessment

**Figure 1:**
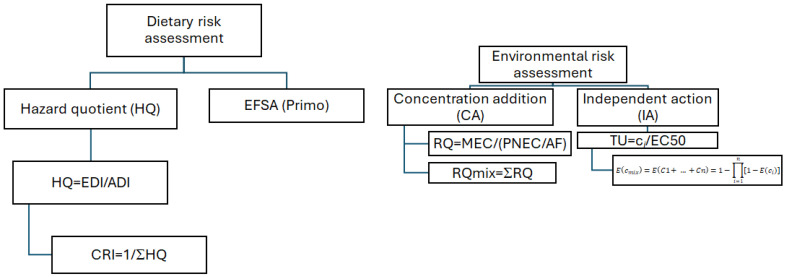
Flowchart of dietary and environmental risk assessment for cumulative contaminations.

The dietary risk assessment was carried out using two approaches: the Hazard Quotient (HQ) method for assessing long-term and acute dietary exposure to chemical contaminants, and the standard tool EFSA PRIMo (Pesticide Residue Intake Model, revision 3.1). This tool consists of an Excel-based calculation spreadsheet that is applied at the EU level in the framework of setting and reviewing maximum residue levels for pesticides under Regulation (EC) No 396/2005 and in the peer review of pesticides under Regulation (EU) No 1107/2009 [5,20,21] (Figure 1). This tool allows for the assessment of both long-term (chronic) and short-term risks (acute). Chronic risk assessment was performed comparing the TMDI (Theoretical Maximum Daily Intake) to the ADI (Acceptable Daily Intake). If the TMDI is lower than the ADI, consumer risk is highly unlikely, and no further dietary risk assessment is needed. For the acute risk assessment, IESTI (International Estimated Short-term Intakes) was compared to the ARfD (Acute Reference Dose). If the IESTI is lower than the ARfD, the acute dietary risk is considered acceptable.

The HQ was determined using the following Equation (3) [22]:

(3) $$\mathrm{Hazard}\;\mathrm{Quotient}\;(\mathrm{HQ})\;=\;HQ=\frac{EDI}{ADI\;or\;ARfD}$$

EDI: Estimated Daily Intake; (mean residue level (mg/kg) × Fi)/Bw.

Fi: median European food consumption data, adult: 0.072 kg/d, toddler (2–3 years): 0.020 kg/d.

Bw: median European body weight (Adult 71.4 kg, toddler 20 kg).

ADI and ARfD: mg/kg bw/d.

TDMI was implemented in EFSA PRIMO using the following equation:

$$TMDI=\frac{\sum_{i=1}^{n}\left(STM\;R_{i}\times C_{i}\right)}{bw}$$

STM R*_i_*: mean residue concentration, calculated from the individual results;

MC_i_: mean consumption for a given RAC;

*bw*: mean body weight for the subgroup of the population related to the LP or mean consumption (in kg).

IESTI was implemented in EFSA PRIMO using the following equation:

$$IESTI=\frac{\left(HR\times v\times LP\right)}{bw}$$

$$\%ARfD=\frac{IESTI}{ARfD}\times 100$$

LP: Large portion reported (in kg/day) (97.5th percentile of eaters (or alternative percentile, depending on the number of reported eating occasions);

HR: Highest residue according to residue definition for enforcement in composite sample (in mg/kg);

*v*: variability factor, depending on the unit weight of the whole product (URAC), for tomatoes is 3;

Adults and toddlers (2–3 years) were considered for HQ risk assessment and the Cumulative risk assessment (CRA). An HQ value > 1 indicates a potential health risk.

Human and mammalian Cumulative Risk Assessment (CRA) was conducted, assessing the Cumulative Risk Index (CRI) as the reciprocal of the sum of the HQ values of each pesticide in the mixture of interest (Hazard Index, HI) [23,24]. The CRI can be calculated considering the NOAEL or ADI values [24]. In this paper, to be consistent with the single pesticide residues’ long-term dietary risk assessment for chemical contaminants evaluation, the ADI was used. When the CRI is greater than 1, the combined risk is considered acceptable.

The cumulative environmental risk assessment was executed using the Concentration Addition (CA) and Independent Action (IA) methods (Figure 1). The Concentration Addition model assumes similar modes of action among the mixture components and is calculated by the sum (RQmix) (5) of the single risk quotient (RQ) (4) calculated by dividing the measured environmental concentration (MEC or c*_i_*) by the predicted no-effect concentration (PNEC) selected as the lowest acute environmental concentration (worst case) and dividing it by a nominative safety assessment factor (AF), or considering the toxic unit (TU) as the environmental concentration for the EC50 (TU = c*_i_*/EC50), respectively. For the calculation made in this paper, the EC50 values for *Daphnia magna* were selected (Table S2), as they are the most sensitive. The risk associated with the mixture was evaluated as high risk (RQmix ≥ 1), moderate risk (0.1 ≤ RQmix < 1), low risk (0.01 ≤ RQmix < 0.1), or rare risk (RQmix < 0.01) [25].

(4) $$RQ=\frac{MEC}{PNEC/AF}$$

RQmix = ΣRQ(5)

Considering the alternative approach to independent Action (IA), E(c_mix_) (6) is the sum of the independent events, considered as a toxic unit (TU).

(6) $$E\left(c_{mix}\right)=E\left(C1+\ldots +Cn\right)=1-\prod_{i=1}^{n}\left[1-E(c_{i})\right]$$

E(c_mix_) is the IA-expected overall effect (scaled to the range 0–1) of a mixture composed of n chemicals, and E(c*_i_*) gives the effect of chemical *i* if applied singly in a concentration c*_i_*.

The Independent Action model assumes dissimilar modes of action and estimates the combined effect as the probability that at least one component causes an effect, assuming independent action.

The maximum cumulative ratio (MCR) was calculated by comparing the toxicity of the mixture to the highest toxicity of a single component (7) [26].

MCR = Toxicity of the mixture/Highest toxicity of a single mixture component.(7)

## 3. Results and Discussion

### 3.1. Method Validation

**Figure 2:**
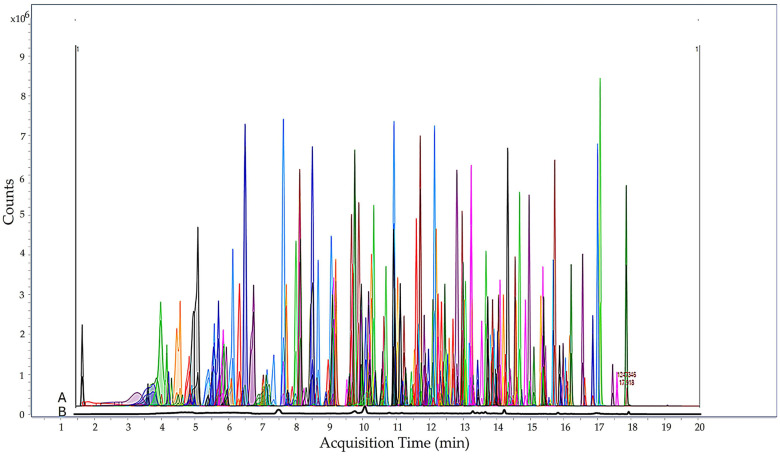
MRM chromatogram of tomato matrix fortified at LOQ with 252 pesticides (A) and of a blank tomato matrix (B).

No interfering peaks were observed within the retention time window of the pesticides for both quantification and confirmation ions, showing acceptable specificity, and no additional cleanup step was necessary (Figure 2). The instrumental response of pesticides was affected (10.3%) by coextracted compounds from the tomato matrix. Among pesticide residues, 64.3% were suppressed, and 35.7% increased, with a range of −52.5% (invermectin B1) to 173.6% (fuberidazol). According to SANTE criteria, the matrix effect in the range of −20 < ME% < 20 was considered insignificant [27].

To compensate for the matrix effect (ME), five-point calibration curves were prepared in blank matrix extracts. Linearity was above the threshold required by the method validation guidelines [18], with correlation coefficients (r^2^) ranging from 0.9959 to 1.0000, and a maximum RSD% of 1.96 (Table S1). The instrument limits of quantification (LOQs) were far below the default MRL set by the European Commission (0.01 mg/kg) [28], with values ranging from 0.002 mg/kg for beflubutamid to 0.007 mg/kg for ivermectin B1a (Table S1). Recoveries ranged from 65.10 to 119.53% at the LOQ level, and from 65.28 to 119.56% at 10xLOQ. However, only the 9% and the 3%, respectively, were below the 70% recovery in agreement with SANTE principles (Table S1) [18]. Repeatability (RSDr) and reproducibility (RSDwR) yielded average results below 12.82%, with maximum and minimum RSD% values of 19.27% and 0.41% in RSDr, and 13.17% and 0.45% in RSDwR. The expanded measure of uncertainty showed values ranging from 3.1% (thiocarb) to 35.8% (flubendiamide), always below 50% of the default values for both spiking levels for all pesticides.

### 3.2. Monitoring Assessment of Pesticide Residues in Tomato Samples

**Figure 3:**
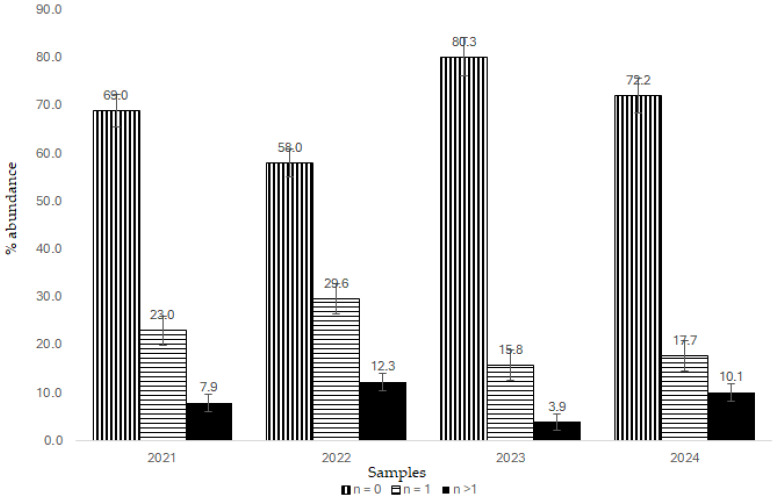
Samples (%) contaminated with pesticide residues during the four-year monitoring period. n = number of pesticide residues detected.

The main tomato adversities requiring the use of plant protection products in Sardinia are represented by the yellow noctule (*Heliothis armigera*), moth (*Tuta absoluta*), downy mildew (*Phytophtora infestans*), and powdery mildew (*Leveillula taurica*). Therefore, fungicides and insecticides are used by farmers to prevent plant damage and fruit losses. Despite the high number of compounds available in the market, in Sardinia, farmers use only a limited number of pesticides, following integrated production regulations and applying techniques for preventing and monitoring pest attacks. Agronomy experts assist farmers in using biological pest control methods, appropriate cultivation techniques, and plant protection products that pose less risk to human health and the environment. Among the pesticides used, on average in all monitored years, 47% were insecticides, 38% fungicides, and 15% herbicides. The most used were chlorantraniliprole, spinosad, fenpyroximate, hexythiazox, emamectin, lambda-cyalothrin, and etofenprox, whereas among fungicides were cymoxanil, penconazole, metalaxyl-M, propamocarb, pyraclostrobin, and mandipropamid. Quizalofop-ethyl and pendimethalin were the most used herbicides. The analysis revealed the presence of 22 pesticide residues in the four years, comprising 68.2% fungicides, 27.3% insecticides, and the remaining 4.5% acaricides; no herbicide was recorded during the analysis. Sample contamination varied over the years, with a positive trend in the last two years, and with an increasing number of samples free from residues (Figure 3). All samples showed residue values below the MRL set on tomatoes; higher levels were detected for Boscalid in 2022 with an average value of 0.42 mg/kg. Overall, the high number of fields sampled and the distinct locations with different phytosanitary problems at different periods of maturity resulted in a very varied and uneven distribution of residues. Most of the contaminated samples had a single pesticide residue (n = 1). The number of single pesticide residues detected was similar in the four years, ranging from 12 (2021) to 19 (2022). The most significant difference was that the maximum levels of pesticide residues showed a downward trend from 2022 to 2024.

Multi-residue contamination occurred each year for a total of 42 different mixtures in the four years; the lower abundance was detected in 2023 (3.9%), and the higher in 2022 (12.3%) (Figure 3).

The maximum number of residues detected in one sample was 5 in 2022. In 2021 and 2022, the mixtures with 3 pesticide residues showed a similar abundance (50%) and decreased till 2024 (25%), whereas those with 2 residues were higher in 2023 (66.7%) and 2024 (75%) (Figure S1). Tomatoes for industrial processing are harvested during the summer from the last week of July to the first week of September. Throughout the period under review, the average temperature was similar in all years, ranging within 20–35 °C, whereas rains were registered at the beginning of August 2021 and in the middle of August 2023. This fact can explain the higher number of fungicide treatments in these years, due to the favourable climatic conditions that allowed for the development of fungi and moulds. A progressive improvement in farming practices and activities related to the selection of less persistent and less toxic pesticides had a positive impact on crop management, increasing tomato safety.

The presence of multiresidue contamination raises concerns about potential cumulative and synergistic effects, requiring the evaluation of a comprehensive risk assessment of the interaction among pesticide residues below the MRL [5,29].

Previous monitoring studies in Morocco on tomatoes analysed 39 samples from the market, searching 432 pesticide residues. The analysis showed residues mainly below the MRL, and multiresidue contamination was detected with a maximum of 7 pesticides in a single sample, but only in one case above the MRL. The compounds detected at a higher frequency were azoxystrobin, spinosad and difenconazole [14]. A different trend was highlighted in a monitoring study in Egypt, showing the presence of 15 pesticide residues with concentrations above the MRL. However, the analytical method applied did not allow the determination of nonvolatile pesticides, the number of pesticide residues investigated was limited to 19 compounds, and the samples analysed were only 18 tomatoes and 18 cucumbers [15]. Another survey made in Morocco in the years 2018–2019 analysed 202 pesticides in 51 samples and showed the presence of 69% of compounds contaminated with residues, always below the MRL [30]. A monitoring study performed in Italy in the years 2020–2021 on fruits and vegetables from the market showed that the 25.8% of the samples had detectable residues, always below the MRL. The most present were tebuconazole and boscalid [31]. From 2013 to 2015, a survey was performed in China on 132 samples of peaches collected from the field. A total of 105 pesticides were searched in the fruits, leading to 39 residues detected. The major number of residues were insecticides, followed by fungicides and acaricides, around 0.6% of contaminated fruits had residues exceeding the MRL. The 76.3% showed multiple residues, with the main mixture composed of two pesticides. The highest number of pesticides detected in a single sample was 13, and the most found were carbendazim, chlorpyriphos, acetamiprid, cypermethrin, and imidacloprid [32]. The official control system of the Italian Ministry of Health in 2020 reported that samples of tomato with detectable pesticide residues were around 50%, with only 0.8% with residues above the MRL. The irregular samples had residues of deltamethrin and chlorfenapyr. In the period between 2015 and 2020, tomatoes were the most sampled crops, and the principal fruits were irregular samples [13]. Data from monitoring surveys on tomatoes for industrial processing are not easily available in the literature, as they are primarily collected by companies for internal control purposes.

In the present survey, most of the residues found in different years were fungicides; in 2021 they were spinosad, mandipropamid, tebuconazole, metalaxyl, zoxamide, and ametotractin; in 2022, and almost all pesticide residues were detected with comparable abundance, even if pyraclostrobin, and zoxamide had the highest frequency. In 2023, the ten pesticides detected in several mixtures showed residues in a maximum of two mixtures; however, in 2024, acetamiprid, azoxystrobin, and spinosad were the most represented. Overall, the total number of residues decreased each year from 2021 to 2024 (Figure S1).

The pesticides found act through different modes of action (MoA); they are all characterised by a low acute toxicity in mammals and no significant carcinogenic, genotoxic, or teratogenic potential at environmentally relevant exposure levels.

Comparing the data with available monitoring studies, the analysis showed fewer samples contaminated with pesticide residues, a reduced number of mixtures, and a lower number of residues for each single sample.

### 3.3. Risk Assessment

The HQ approach is a first-tier empirical risk assessment method used to assess acute or chronic risk [24]. The EDI_adult_ values ranged from 1.01 × 10^−5^ to 3.49 × 10^−4^ mg/kg bw/d, whereas EDI_toddlers_ ranged from 1.01 × 10^−5^ to 3.44 × 10^−4^ mg/kg bw/d. All HQ values were <1, indicating that the single pesticide residues detected in tomato samples did not pose any health risk for consumers. Emamectin exhibited the highest HQ value, accounting for 4.51 × 10^−2^ and 4.45 × 10^−2^ for adults and toddlers, respectively (Table 1).

Chronic risk assessment carried out with the EFSA’s PRIMo tool showed ADI% ranging from 0% to 36% for adults and the general population (Table S2) and from 0% to 10% for children, toddlers, and infants (Table S3). As none of the residues exceeded 100% of the ADI, no chronic risk for consumers is expected.

Acute risk assessment performed with the HQ showed values below 1, indicating that the single pesticide residues detected in tomato samples did not pose any acute health risk to consumers. Azoxystrobin, mandipropamid, zoxamide, clofentezin, and ametotractin reported an insignificant ARfD in the EFSA conclusions, and the acute risk was not assessed (Table S4).

Acute risk assessment carried out with the EFSA’s PRIMo tool showed ARfD% ranging from 0.1% to 45.9% for adults and from 0.2% to 55.8% for toddlers, not posing any acute health risk to consumers. However, when the new ARfD value for acetamiprid (0.005 mg/kg bw/d) was entered into the system, the ARfD% increased to 125.4%, exceeding the safety value of 100% (Table S5) for BE children with a daily intake of tomatoes of 180 g, whereas data from HQ calculations for an edible portion of 20 g led to HQ < 1. New considerations should therefore be made for the single compound acetamiprid, regarding the food daily intake (FDI) for a selected group of consumers.

IESTI values indicate that, based on the detected residual levels, there is no short-term risk to adults or toddlers from the pesticides evaluated in the present study. Although all IESTI remains below the established ARfDs (Table S6), some substances contribute significantly to the overall exposure, particularly in toddlers (Table S4).

Researchers and legislators mainly assess the safety of chemicals one at a time. When the potential toxicological risk is below the reference value for acute and chronic toxicity, the use of the pesticide is considered acceptable. However, the new trend in risk assessment is to address chemical mixtures of toxic compounds. Consumers are exposed daily to a high number of toxic compounds at the same time or over a restricted period. Among these, there is a high probability of being exposed to more than one pesticide within the same food or food product. The EFSA’s latest report, published in 2023, reported the data from the European Union, with 58.1% of samples free from detectable pesticides, but 39.8% containing one or more pesticide residues below the MRL [33]. Combined exposure can occur with pesticides present in the same composite samples containing multiple residues or from combined foods containing one or more pesticide residues. Furthermore, exposure can occur through different pathways, such as oral, inhalation, or dermal, and each of these is considered independently, even if it is the totality of the exposure that determines the real risk. In this paper, we considered the pesticide residue in tomato samples, and therefore, the only route of contamination is oral. Moreover, it is a single ingestion, and chronic assumption of the mixtures detected is unrealistic.

Whatever the possibility of encountering multiple contaminations, the interaction among PPPs can follow different patterns: dose-addition, response-addition, synergism, or antagonism. Therefore, understanding the effective toxicity of a mixture is a challenging task. The assessment of the cumulative dietary exposure to multiple pesticide residues is in line with the EFSA and EPA Commission guidelines for cumulative risk assessment (CRA), which deal with the correct approach to define a risk assessment when more than one toxic is found in food commodities. The EFSA recommends that cumulative risk assessments for pesticides consider all the substances that belong to the common mechanism group (CMG), with a similar MoA when exposure occurs through the consumption of the same food commodity. Regarding the interaction among specific pesticides at low, non-effective doses, a case-by-case approach should be put in place [23].

The correct criteria to be applied for cumulative risk assessment should consider several issues and could be adapted to different situations, due also to the availability of toxicological data of different natures. When performing human risk assessment, the mechanism of mammalian toxicity (concerning human and animal health), exposure is assessed by combining occurrence data on chemicals with consumption data, whereas concentration data are commonly used for environmental and ecological evaluations. The occurrence and representative concentrations of PPPs residues in food commodities should be used for risk assessment; however, these data are few, and even if not perfectly suitable, data from supervised or monitoring trials can be used. When the components of the mixture and their exposure levels are chemically defined, it is possible to apply the component-based approach with exposure and hazard data of the individual components.

Since the pesticide concentrations found were highly variable in the different mixtures and in many cases close to the LOQ, biotesting is not a viable path, and offline modelling approaches should be considered for risk assessment purposes of the mixtures.

First, the PPP’s chemical and toxicity activities were defined to set the appropriate assessment group of the mixture. The pesticide detected in the mixture belonged to various categories: fungicides (68.2%), insecticides (27.3%), and acaricides (4.5%). The toxicities of the pesticides included in the mixtures for humans and other mammals were classified between II and IV according to the Environmental Protection Agency (EPA), with 80% Class III and IV having a moderate to low toxicity (Table 2).

The toxicity of the compounds detected in the mixtures acts at distinct districts, including the mitochondria, the endocrine system, and energy production, through the inhibition of the cytochrome bc1 complex in the mitochondria. However, most of them have specific activity on insect and fungal biochemical mechanisms, which do not overlap with human ones.

Therefore, grouping the pesticides in mixtures for their MoA is not an easy task. In this paper, we decided to consider all pesticides in the mixtures without considering the MoA, even if this could lead to an overestimation of the cumulative effect.

The analysis of the pesticide mixture showed no critical concern for human safety. All mixtures had CRI values well above the baseline value of 1, ranging from 16.4 to 578.0 in adults and from 26.1 to 581.4 in toddlers (Table 3).

The pesticides that contributed most to the HQ values were spinosad, boscalid, and pyraclostrobin in 2021, spinosad and pyraclostrobin in 2022, acetamiprid, tebuconazole, and pyraclostrobin in 2023, and acetamiprid, metalaxyl, tebuconazole, and spinosad in 2024.

Currently, the European environmental risk assessment of pesticides (ERA) [20] considers almost exclusively single applications of single PPP on a single crop. This means that ERA considers only a mixture of single pesticides present in the same PPP, which could be a formulation of one or more pesticides and different additives. Therefore, no assumptions are reported by ERA regarding uncontrolled pesticide mixtures present in the environment. The literature data confirmed the absence of a regulation in any part of the world regarding this last issue [34]. For environmental risk assessment of a single compound, the Predicted Environmental Concentration to the Predicted No-Effect Concentration (PEC/PNEC) ratio, the Toxicity Exposure Ratio (TER), or a specified Margin of Safety factor could be applied. It is hypothesised that by safeguarding the most sensitive trophic level, all other organism groups are concomitantly protected, and that protecting the structure of an ecosystem also protects ecosystem functions. Further data on environmental exposure or hazard may be needed to clarify the environmental risk, depending on the initial risk assessment’s results. These data are generated from a series of studies known as higher-tier studies, which are becoming more comprehensive. A relevant comparison must be made at each tier between the estimated exposure and the estimated hazard. There are, therefore, separate tiers for both exposure and hazard estimation. However, it should be considered that when environmental toxicity is considered, different targets should be evaluated during ecotoxicological assessments, such as birds and mammals, terrestrial vertebrates from wildlife, bees and other arthropods, and aquatic organisms. The EFSA approach to aquatic organisms’ claims for a 10-step decision tree comparing measured vs. calculated product toxicity to check for a toxicity driver, synergism, and/or antagonism [35]. However, the aquatic guidance assumes that “observed effects are, in many cases, related to the effects of one or two pesticides”. Aquatic organisms’ calculations start using the FOCUS stepwise procedure, which allows for defining a risk profile and mitigation measures. For each pesticide in the mixture, the most sensitive aquatic taxa can be different; all these variables require hundreds of calculations. Therefore, alternative strategies based on worst-case risk quotients (RQs) can be more easily applicable, providing sufficiently reliable information on toxicity.

In this study, the measured pesticide concentrations in tomato matrices were assumed to be equal to those released in surface water. However, this is a worst-case assumption that does not consider the dilution effect or degradation in the water medium. This strategy was adopted due to the varying environmental conditions in the field, with the capacity of rivers and ponds changing rapidly with elevated summer temperatures and reduced rainfall.

The idea behind a mixture risk assessment is to use evaluation variables from earlier studies so that unnecessary experiments can be avoided. Therefore, toxicity calculations can be based on established reference models: Concentration Addition (CA) (also known as dose addition) and Independent Action (IA) (also known as response addition) [25,36,37,38].

Concentration Addition by RQmix showed mainly moderate risk (50.0%), and 25% had a low or a high-risk level (Table 4). Risk quotients provide an immediate understanding of (un)acceptability of a risk without additional information (AF or acceptability criteria), giving a worst-case aquatic risk assessment; the sum can be directly used for a risk assessment of the mixture. The theory behind IA is that the chemicals in a mixture do not physically, chemically, or biologically interact; therefore, some doubt exists considering the use on living organisms. Nevertheless, it has been discovered to offer reliable predictions of chemical mixtures with varying sites or modes of action in test systems comprising algae and bacteria [39].

IA and CA predictions are similar, with some pesticides having a higher weight in driving the toxicity value. CA was the most conservative and usually predicts a higher mixture toxicity [40].

Pyraclostrobin and etofenprox were the main pesticides responsible for the RQ and IA values ≥ 1 in all years except in 2024, in which the moderate risk in CA was attributed to azoxystrobin.

Pyraclostrobin is a synthetic fungicide that acts as a quinone outside inhibitor (QoI) that targets fungal respiration by inhibiting the cytochrome bc1 complex. The NOAEL for short-term oral toxicity is 6 mg/kg BW, the acceptable daily intake (ADI) is established at 0.03 mg/kg bw/d, and the acute AOEL (AAOEL) is established at 0.015 mg/kg bw, on the same basis as the ARfD, applying the AF of 100 and a correction for oral absorption value of 50%; pyraclostrobin is also classified as STOT RE 212 based on effects in liver, gastrointestinal tract and the nasal cavity through prolonged or repeated exposure.

Pyraclostrobin has been associated with adverse effects on neuronal proliferation and potential endocrine-disrupting activity, particularly anti-androgenic effects [41,42]. These effects may contribute to a common outcome of neurodevelopmental impairment, justifying their inclusion in cumulative assessment groups (CAGs) targeting the nervous system.

Etofenprox is an insecticide pyrethroid derivative, and it disturbs insect nervous systems following direct contact or ingestion. The acceptable daily intake (ADI) of etofenprox is 0.03 mg/kg bw/d based on the long-term mouse study and applying an AF of 100, which is supported by the long-term rat study. The acceptable operator exposure level (AOEL) is 0.06 mg/kg bw/d based on the oral 90-day rat study, applying an AF of 100 and a correction factor of 30% for low oral absorption. The acute reference dose (ARfD) was set at 1.0 mg/kg bw, based on the NOAEL of 100 mg/kg bw/d from the rabbit developmental toxicity study and an AF of 100. The available acute toxicity data for etofenprox suggested a classification as very toxic to aquatic organisms, based on the acute toxicity to fish (LC50 = 2.7 µg a.s./L), daphnia (LD_50_ = 1.2 µg a.s./L), and algae (EC_50_ > 150 µg a.s./L).

The environmental impact of the mixture is calculated based on the residues found in tomato samples and leads to values that would suggest a negative impact on aquatic organisms. However, considering the average tomato amount per hectare (75 tons), multiplying by the amount of pyraclostrobin or etofenprox for kg of tomato, the residues left in the field will be almost ¼ of the application dose reported on the label.

The IA evaluation seems to be more efficient in describing the environmental toxicity with respect to CA for the pesticides in the mixtures. This fact was probably related to the dissimilarity of the MoA, which represents a valid criterion for selecting the most appropriate concept.

The MCR values were always ≥1, indicating a contribution to the toxicity of the mixture, only slightly higher than the single compound with the highest toxicity.

## 4. Conclusions

Numerous risks are faced by both humans and wild animals, including environmental pollutants via multiple exposure pathways. The negative impact of a single chemical is sometimes disregarded because its concentration in the environment is so low. The issue of mixture toxicity has become a hot topic, especially when it comes to the additive and synergistic effects of environmental mixtures. The present monitoring survey allowed the assessment of tomato contamination by pesticide residues over four years, from 2021 to 2024. This study remarks that when PPP treatments are made following the label, with field observation for treatment decisions, and using PPPs with low toxicity, the residues are not only below the MRL but are near the instrumental limit of determination.

The samples showed a higher presence of contamination from a single compound rather than contamination from multiple residues. The high number of fields sampled in different areas with different pest problems has led to dissimilar pesticide mixture contamination. The consumer risk assessment carried out with the HQ method and EFSA-PRIMO identified no potential health concerns for adults or toddlers, and the combined risk was considered acceptable, except for acetamiprid in 2024, which changed the ADI from 0.025 to 0.005, leading to an IESTI value of 115% of the ARfD.

The MoA of the pesticides detected was different among the mixtures. Nevertheless, the environmental risk assessment was carried out both by CA and IA modes. CA showed values in some cases too high, whereas IA may provide a more realistic estimate given the likely dissimilar modes of action. The EFSA suggests using CA when pesticides can be included in the same cumulative assessment group; however, this strategy exhibits many deficiencies when pesticide mixtures are constituted by many different pesticides with different MoA. In contrast, IA does not have a solid foundation of scientific research to validate its use in these cases.

The study has some limitations related to several factors, including the actual deposition of residues and the extent of their dilution in surface water. The number of pesticides analysed is consistent with the agricultural production in the areas considered (tomatoes, artichokes, melons, and fennel) but does not account for any unauthorised use of pesticides on other horticultural crops or the metabolites formed after treatments. Furthermore, it does not take into account soil contamination and possible leaching into groundwater. Regarding surface water, the primary critical issue is the inability to make accurate estimates of dilution due to the significant variability of watercourses and ponds near crops during the summer season. However, it should be noted that the assessment of the risks of pesticides to aquatic invertebrates in the assessment dossiers does not take into account dilution or degradation. The definition of correct methods for assessing the dietary risk and the environmental risk of pesticide mixtures at concentrations below the MRL is still in its infancy, due to the difficulty of verifying an additive or synergistic effect, or an unpredictable effect of the mixture. Furthermore, the variability in the composition of mixtures represents an additional gap that is difficult to resolve. The implementation of bioassays to validate IA predictions and computational systems capable of simultaneously assessing different pesticides with different MOAs could be the most effective solution. Moreover, a deeper study should be performed on the fate and risk assessment of the degradation products.

## Figures and Tables

**Table 1:** Consumer chronic risk assessment based on the HQ approach.

		Adults		Toddlers	
Pesticide	ADI (mg/kg/d)	EDI (mg/kg bw/d)	HQ	EDI (mg/kg bw/d)	HQ
Acetamiprid	0.005 *	1.26 × 10^−4^	2.53 × 10^−2^	1.25 × 10^−4^	2.49 × 10^−2^
Acetamiprid	0.025	1.26 × 10^−4^	5.05 × 10^−3^	1.25 × 10^−4^	4.98 × 10^−3^
Cymoxanil	0.013	5.39 × 10^−5^	4.15 × 10^−3^	5.32 × 10^−5^	4.09 × 10^−3^
Metalaxyl	0.08	6.48 × 10^−5^	8.11 × 10^−4^	6.39 × 10^−5^	7.99 × 10^−4^
Azoxystrobin	0.2	1.18 × 10^−4^	5.92 × 10^−4^	1.17 × 10^−4^	5.84 × 10^−4^
Boscalid	0.04	3.49 × 10^−4^	8.73 × 10^−3^	3.44 × 10^−4^	8.61 × 10^−3^
Mandipropamid	0.15	3.77 × 10^−5^	2.51 × 10^−4^	3.71 × 10^−5^	2.48 × 10^−4^
Dimethomorph	0.05	1.02 × 10^−4^	2.04 × 10^−3^	1.01 × 10^−4^	2.02 × 10^−3^
Myclobutanil	0.025	4.06 × 10^−5^	1.62 × 10^−3^	4.00 × 10^−5^	1.60 × 10^−3^
Tetraconazole	0.004	5.58 × 10^−5^	1.39 × 10^−2^	5.50 × 10^−5^	1.38 × 10^−2^
Penconazole	0.03	2.49 × 10^−2^	9.37 × 10^−4^	2.77 × 10^−5^	9.24 × 10^−4^
Tebuconazole	0.03	4.09 × 10^−3^	3.94 × 10^−3^	1.17 × 10^−4^	3.89 × 10^−3^
Zoxamide	0.5	7.99 × 10^−4^	2.15 × 10^−4^	1.06 × 10^−4^	2.12 × 10^−4^
Spinosad (sum A + D)	0.024	5.84 × 10^−4^	3.10 × 10^−3^	7.34 × 10^−5^	3.06 × 10^−3^
Pyraclostrobin	0.03	8.61 × 10^−3^	2.11 × 10^−3^	6.25 × 10^−5^	2.08 × 10^−3^
Clofentezin	0.017	2.48 × 10^−4^	2.00 × 10^−3^	3.35 × 10^−5^	1.97 × 10^−3^
Difenoconazole	0.01	2.02 × 10^−3^	2.33 × 10^−2^	2.30 × 10^−4^	2.30 × 10^−2^
Ametoctradin	10	1.60 × 10^−3^	1.07 × 10^−5^	1.06 × 10^−4^	1.06 × 10^−5^
Metaflumizone	0.01	1.38 × 10^−2^	1.01 × 10^−3^	1.00 × 10^−5^	1.00 × 10^−3^
Emamectin	0.0005	2.49 × 10^−2^	4.51 × 10^−2^	2.23 × 10^−5^	4.45 × 10^−2^
Etofenprox	0.03	4.04 × 10^−5^	1.35 × 10^−3^	3.98 × 10^−5^	1.33 × 10^−3^

* ADI changed in 2024.

**Table 2:** Toxicological and chemical characteristics, together with the main uses of the pesticides detected in the mixture.

Plant Protection Product	Toxicity Class (EPA)	NOAEL mg·Kg bw·d	Toxicity Activity	Chemical Class	Main Uses
Acetamiprid	II/III	10	Acetylcholine, nicotinic receptors	neonicotinoid	insecticide
Cymoxanil	II	0.01	Inhibits cytochrome c oxidase in the fourth complex of the electron transport chain	organic nitriles	fungicide
Metalaxyl	III	9.4	Disrupts fungal nucleic acid synthesis by inhibiting RNA polymerase I	phenylamide	fungicide
Azoxystrobin	III/IV	20	Quinone outside inhibitor (QoI)-type	strobilurin	fungicide
Boscalid	III/IV	22	Mitochondrial damage	aromatic anilides	fungicide
Mandipropamid	IV	247.6	Inhibitor of phospholipid biosynthesis	mandelamide	fungicide
Dimethomorph	III	16	Endocrine disruptor	morpholine group	fungicide
Myclobutanil	II	2.5	Induce liver microsomal enzymes	triazole	fungicide
Tetraconazole	III/IV	2.95	Interact with nuclear receptors (constitutive androstane receptor, CAR; pregnane X receptor, PXR), liver toxicity.	triazole	fungicide
Penconazole	III	69	Increase oxidative damage and modify enzyme activity	triazole	fungicide
Tebuconazole	III/IV	3	Activate nuclear receptors (aryl hydrocarbon receptor, AHR), trigger oxidative stress and apoptosis.	triazole	fungicide
Zoxamide	III/IV	50	Mitotic arrest by disrupting cell division, binding to β-tubulin and inhibiting tubulin polymerisation	benzamide	fungicide
Spinosad (Sum A + D)	III	4.89	Nicotinic acetylcholine receptor	spynosin	insecticide
Pyraclostrobin	IV	3.4	Quinone outside inhibitor (QoI)-type	strobilurin	fungicide
Clofentezin	III	1.95	Effect on the liver (enzyme induction) Affects thyroid function and impairs steroid hormone regulation	tetrazine	acaricide
Difenoconazole	III/IV	3.7	Disruptions to the endocrine system and nervous system	dioxolanes	fungicide
Ametoctradin	IV	1000	Disrupting mitochondrial energy production	triazolopyrimidine	fungicide
Metaflumizone	IV	40	Sodium channel blocker	semicarbazone	insecticide
Emamectin	II	0.6	Chloride channel activator by binding gamma aminobutyric acid (GABA) receptor and glutamate-gated chloride channels disrupting nerve signals within arthropods	second-generation avermectin	insecticide
Etofenprox	IV	23	Disturbs insect nervous systems following direct contact or ingestion	pyrethroid derivative	insecticide

**Table 3:** Cumulative risk assessment for adults and toddlers for the pesticide mixtures detected in the survey.

	2021	2022	2023	2024
Residues (mg/kg ± RSD%) *				
min	0.058 ± 3.61	0.068 ± 5.59	0.067 ± 5.27	0.104 ± 9.17
max	0.228 ± 3.80	1.061 ± 1.27	0.584 ± 9.77	0.403 ± 5.73
median	0.133 ± 11.46	0.204 ± 2.46	0.188 ± 1.96	0.260 ± 1.53
CRI adult				
min	22.64 ± 16.03	21.90 ± 1.26	81.12 ± 9.03	54.14 ± 13.84
max	3096.03 ± 20.10	879.30 ± 9.15	1250.86 ± 5.07	679.97 ± 10.10
median	446.49 ± 4.14	237.70 ± 4.98	177.53 ± 2.63	115.61 ± 4.49
CRI toddler				
min	22.96 ± 16.03	22.21 ± 1.26	82.26 ± 9.03	54.91 ± 13.84
max	3139.64 ± 20.10	891.68 ± 9.15	1268.48 ± 5.07	689.54 ± 10.10
median	452.78 ± 4.14	241.03 ± 4.97	180.03 ± 2.63	117.24 ± 4.49

* Sum of residues of the single mixtures.

**Table 4:** CA and IA environmental risk assessment for the pesticide mixtures detected in the survey.

	2021	2022	2023	2024
CA				
min	0.10	0.01	0.52	0.00
max	14.10	15.36	235.0	0.95
median	0.46	10.93	1.86	0.93
IA				
min	0.01	0.01	0.22	0.00
max	1.37	15.25	5.64	00.095
median	0.51	2.62	1.56	0.093

## Data Availability

The original contributions presented in this study are included in the article/Supplementary Materials. Further inquiries can be directed to the corresponding author(s).

## Supplementary Materials

The following supporting information can be downloaded at https://www.mdpi.com/article/10.3390/jox15050171/s1, Figure S1: Pesticide Frequency; Table S1: Linear regression equations of the new validated pesticides and matrix effects; Table S2: Chronic risk assessment (JMPR methodology—IEDI/TMDI) for EU populations (Adult and General Population) using EFSA’s PRIMo tool revision 3.1; Table S3: Chronic risk assessment for EU populations (JMPR methodology—IEDI/TMDI) (Children, toddler and infant) using EFSA’s PRIMo tool revision 3.1; Table S4: Consumer acute risk assessment based on the HQ approach; Table S5: Acute risk assessment for EU populations (toddler and adults) using EFSA’s PRIMo tool revision 3.1; Table S6: Table of all available data on the acute toxicity to aquatic invertebrates [43,44,45,46,47,48,49,50,51,52,53,54,55,56,57,58,59,60,61,62].
