# Supplementary material for: Four-Year Monitoring Survey of Pesticide Residues in Tomato Samples: Human Health and Environmental Risk Assessment
Source: J Xenobiot. 2025 Oct 20;15(5):171. doi: 10.3390/jox15050171 (PMC12564937; doi:10.3390/jox15050171)
Supplement: Supplementary file 1 [file jox-15-00171-s001.zip › jox-3883729-supplementary/Table S1.pdf]

Table S1. Linear regression equations of the new validated pesticides and matrix effects.

Pesticide	Linear regression equation	r^2 ± RSD%	ME%	LOQ	AR (% ± RSD%)		RSD_r	RSD_{wR}	U
	in tomato matrix			mg·kg^{-1}	LOQ	10xLOQ	RSD%		
Amidosulfuron	y = 10389843x + 19608	0.997±0.33	1.6	0.004	72.60±10.82	67.61±5.25	2.28	3.04	7.60
Aminocarb	y = 72427397x + 119870	0.996±1.14	-10.6	0.003	86.47±14.32	86.73±3.68	0.57	3.65	7.39
Azaconazole	y = 36058676x + 47532	0.999±0.99	-1.2	0.004	86.21±4.40	92.60±1.31	0.46	8.85	17.72
Azamethiphos	y = 47803190x + 48794	0.999±0.90	1.7	0.004	85.03±2.86	93.25±1.62	3.32	12.82	26.49
Beflubutamid	y = 70539069x + 190262	0.996±1.05	-21.0	0.002	84.53±13.66	90.54±3.24	0.50	1.88	3.89
Benzoximate	y = 43040132x + 61845	0.997±0.63	0.2	0.004	83.00±3.93	88.35±2.47	0.86	6.07	12.26
Bromuconazole	y = 2668557x + 5864	0.995±1.75	3.9	0.003	109.34±2.91	92.58±3.55	1.40	8.85	17.92
Butocarboxim	y = 20764089x + 202	0.999±1.32	-3.2	0.005	101.35±5.31	101.07±2.81	2.50	11.73	23.99
Carboxin	y = 54023847x + 41517	0.999±1.14	0.8	0.004	87.41±4.98	93.36±1.37	4.89	4.92	13.87
Carfentrazone-ethyl	y = 7513924x + 16996	0.997±0.74	-3.8	0.004	65.67±9.67	97.17±1.44	6.70	12.71	28.74
Chlorfenvinphos	y = 9097387x + 17630	0.997±0.56	-16.7	0.003	107.91±6.30	98.66±2.64	7.14	7.37	20.52
Chloridazon	y = 21699771x + 24850	1.000±0.24	-2.1	0.005	92.26±6.54	108.59±8.85	8.20	4.84	19.04
Chlorsulfuron	y = 3614674x + -4116	0.999±0.15	4.1	0.005	65.80±6.51	65.45±7.45	5.94	1.72	12.37
Clethodim	y = 7090742x + 12552	0.999±1.15	-9.5	0.005	68.24±10.28	69.78±2.83	8.30	2.31	17.23
Clomazone	y = 49735661x + 121391	0.996±0.46	-1.2	0.004	99.28±3.77	103.37±0.99	10.12	2.82	21.01
Coumaphos	y = 17937488x + 21114	0.998±1.26	-5.2	0.004	94.34±9.17	97.52±2.79	8.46	4.73	19.38
Cycluron	y = 55676863x + 88025	0.998±1.02	0.5	0.004	86.02±5.90	90.69±1.17	7.21	2.68	15.38
Cymiazol	y = 27496404x -11193	1.000±0.93	-6.6	0.004	90.19±24.06	75.92±23.52	6.69	2.93	14.61
DEET	y = 160467199x + 393874	1.000±0.40	2.9	0.004	85.53±2.68	93.77±1.41	19.27	7.44	41.31
Desmedipham	y = 45795366x + 41946	0.999±1.06	-1.9	0.004	89.17±3.36	92.89±0.05	3.73	5.37	13.08
Diazinon	y = 37051404x + 32982	0.999±1.40	3.9	0.004	90.76±2.45	92.86±2.12	6.41	3.94	15.05
Diflubenzuron	y = 8115029x + 32681	0.995±0.49	-3.4	0.003	67.03±22.87	93.80±0.19	5.19	3.24	12.24
Diflufenican	y = 25298356x + 44785	0.999±0.57	1.7	0.005	81.50±16.74	92.75±1.77	10.00	5.54	22.86
Dimethachlor	y = 129424708x + 79594	0.999±0.33	5.7	0.005	86.78±3.50	92.00±1.08	7.60	1.75	15.60
Dimoxystrobin	y = 141165845x + 162816	0.998±1.70	6.7	0.004	92.43±2.37	90.27±2.26	2.65	6.80	14.60
Dioxacarb	y = 36496449x + 60388	0.998±0.27	-2.3	0.004	79.85±5.39	91.55±2.81	10.43	7.64	25.86
Epoxyconazol	y = 22259533x + 6513	1.000±1.51	-1.7	0.004	98.83±7.48	94.99±1.50	0.55	1.58	3.35
Ethidimuron	y = 39033794x + 51388	0.999±0.40	15.3	0.004	85.17±4.92	93.99±1.39	8.88	10.03	26.79
Ethion	y = 39062343x + 43714	0.999±0.73	-6.2	0.005	86.00±9.78	91.24±1.56	3.42	5.98	13.78
Ethofumesat	y = 763710x + 1960	0.997±1.61	-9.6	0.004	103.29±10.21	111.19±5.43	9.40	2.67	19.54
Ethoxyquin	y = 17282654x + 4537	0.999±1.33	1.4	0.004	93.51±5.09	90.80±1.53	3.90	1.78	8.57
Fenazaquin	y = 85777372x -7025	1.000±1.12	-12.4	0.005	88.23±11.98	83.51±7.69	7.77	2.98	16.64
Fenbuconazole	y = 10037121x + 2629	0.999±1.00	1.1	0.005	86.48±1.65	89.23±3.43	2.19	10.03	20.53
Fenobucarb	y = 11963874x + 9914	0.999±1.10	0.4	0.004	98.67±1.43	102.86±2.67	5.41	7.82	19.02
Fenoxycarb	y = 24160939x + 50468	0.998±0.89	0.3	0.004	81.01±8.51	95.56±2.80	4.03	3.00	10.05
Fenpropidin	y = 36051230x + 9778	1.000±0.97	1.8	0.005	85.73±5.90	89.41±0.97	0.41	2.95	5.96
Fenuron	y = 4770221x + 495	1.000±0.93	27.2	0.005	72.42±4.25	81.05±1.17	11.43	2.98	23.62

Fipronil	y = 7586990x + 11853	0.997±0.21	2.6	0.004	81.67±10.23	85.43±3.04	12.73	2.53	25.96
Fluazinam	y = 6469631x + 14553	0.995±0.70	1.4	0.003	78.48±10.03	85.76±1.13	6.62	2.61	14.23
Flubendiamide	y = 1209419x + 3924	0.996±1.63	-30.4	0.003	88.79±35.09	80.65±3.22	17.31	4.55	35.80
Fludioxonil	y = 1278315x + 3043	0.996±1.66	5.5	0.004	82.89±19.93	84.14±3.15	11.38	3.19	23.64
Flumioxazin	y = 218446x + 22	0.996±1.65	-9.9	0.006	87.77±8.72	121.89±4.98	8.48	13.17	31.33
Fluometuron	y = 105173034x + 175301	0.997±0.54	-1.0	0.004	91.14±4.51	95.65±1.26	5.04	1.93	10.79
Fluopicolide	y = 37853167x + 89196	0.995±1.70	-4.3	0.004	86.47±7.29	93.27±2.02	8.63	2.95	18.24
Fluoxastrobin	y = 40776460x + 91370	0.997±1.53	-3.6	0.004	93.32±2.35	101.17±2.91	4.22	8.23	18.50
Fluquinconazole	y = 3934351x + 6578	0.997±1.45	-3.8	0.004	92.00±15.75	95.61±3.70	6.10	3.59	14.16
Forchlorfenuron	y = 18661851x + 23199	0.998±0.28	3.8	0.004	82.46±3.52	89.86±0.42	8.40	8.75	24.26
Fuberidazol	y = 99435592x -354419	0.996±0.88	173.6	0.006	113.49±18.01	65.28±5.07	8.27	1.00	16.66
Furalaxyl	y = 125914907x + 275144	0.996±1.13	5.8	0.004	82.80±1.79	90.93±1.98	3.46	3.23	9.47
Furathiocarb	y = 27585269x + 12260	1.000±1.37	4.0	0.005	84.19±7.93	89.74±1.19	8.33	6.37	20.97
Halofenozide	y = 5996623x + 17562	0.995±1.81	5.4	0.004	68.82±8.23	83.01±1.34	3.85	2.77	9.49
Halosulfuron-methyl	y = 5158416x + -2819	0.999±1.65	1.3	0.005	73.29±9.58	71.08±7.26	5.11	1.69	10.76
Hexaconazole	y = 9235014x + 10540	0.999±0.48	2.3	0.004	91.64±5.08	93.60±0.99	1.65	2.02	5.22
Hydramethylnon	y = 38792829x + 26972	0.998±0.93	2.5	0.004	85.24±4.06	83.67±0.38	3.40	8.00	17.39
Ipconazole	y = 27080346x + 21047	1.000±0.58	1.5	0.005	84.09±9.36	88.61±0.63	7.28	1.35	14.81
Isocarbophos	y = 30069150x + 49188	0.998±1.12	-7.0	0.004	93.81±5.50	100.57±0.60	2.65	10.20	21.08
Isofenphos-methyl	y = 5284779x + 2117	1.000±1.82	4.0	0.005	81.99±6.26	92.72±4.14	5.19	1.75	10.95
Isoprothiolane	y = 148262104x + 276652	0.997±1.20	0.1	0.004	91.91±4.40	94.62±0.47	2.98	1.03	6.31
Isoxaben	y = 90186824x + 84777	0.999±0.58	2.2	0.005	90.92±3.19	92.82±2.51	1.53	6.55	13.45
Isoxaflutole	y = 1231943x + 1344	0.997±0.07	-6.9	0.004	107.97±11.73	99.09±6.73	2.65	2.27	6.98
Ivermectin B1a	y = 34024x -22	0.998±1.89	-52.5	0.007	119.53±6.98	119.56±0.08	10.30	5.02	22.92
Kresoxim-methyl	y = 10822277x + 24526	0.996±0.25	-3.2	0.004	89.04±2.55	94.03±0.56	5.20	0.45	10.44
Lenacil	y = 12225376x + 19555	0.998±1.11	-21.3	0.004	118.51±5.46	118.00±1.46	5.76	2.66	12.69
Linuron	y = 14494667x + 17845	0.999±1.36	-1.2	0.004	82.98±5.55	95.78±2.06	8.62	1.25	17.42
Malaoxon	y = 61129757x + 54528	0.999±0.88	2.2	0.004	86.67±2.35	93.10±2.60	2.61	12.51	25.56
Malathion	y = 13001471x + 38693	0.995±0.60	-0.1	0.003	66.15±7.83	86.35±4.17	4.09	1.31	8.59
Mecarbam	y = 24495951x + 28477	0.997±1.90	6.9	0.004	84.71±4.66	86.92±1.20	7.23	5.80	18.54
Metamitron	y = 12804931x + 12173	0.999±0.08	-4.1	0.004	84.40±3.85	93.75±2.66	3.70	2.18	8.59
Metazachlor	y = 65829660x + 71566	0.999±0.52	1.6	0.004	90.31±5.66	93.02±0.83	3.19	4.39	10.85
Metconazole	y = 21581178x + -3454	1.000±0.47	0.8	0.005	89.86±8.41	88.93±2.23	0.80	2.57	5.38
Methabenzthiazuron	y = 157716069x + 193312	0.999±0.07	1.9	0.004	114.74±4.89	112.68±1.48	5.31	3.31	12.51
Methacrifos	y = 3192811x + 10643	0.995±0.87	-2.4	0.004	69.05±5.84	102.77±1.15	4.40	7.50	17.39
Methoprotryne	y = 60230580x + 33554	0.999±1.07	1.2	0.004	91.73±3.64	90.35±0.50	1.13	2.52	5.52
Methoxyfenozide	y = 25200809x + 24396	0.999±0.68	-2.5	0.005	87.18±2.44	100.24±0.89	10.55	7.09	25.42
Metobromuron	y = 18629944x + 31472	0.998±0.61	-3.2	0.004	85.84±3.46	95.34±0.88	4.15	7.76	17.60
Metolachlor	y = 93251470x + 64978	0.999±1.26	1.9	0.004	92.65±4.49	90.68±1.97	2.74	6.85	14.76
Mevinphos	y = 15699442x + 13065	0.999±1.23	1.3	0.004	86.72±3.97	95.08±3.39	4.70	2.52	10.67
Mexacarbate	y = 106716855x + 13670	1.000±0.92	-1.0	0.005	87.06±3.33	91.59±2.48	12.61	4.35	26.68

Moxidectin	y = 480738x + -32	0.999±1.90	-16.1	0.004	89.10±1.08	70.10±1.45	9.96	5.96	23.21
Nitenpyram	y = 8496991x -469	1.000±0.62	-10.7	0.005	76.71±32.42	98.66±8.75	5.41	3.37	12.75
Novaluron	y = 4660646x + 3623	0.999±0.53	5.2	0.006	75.24±16.92	87.35±3.24	8.50	8.69	24.31
Oxadixyl	y = 15458339x -5662	1.000±0.59	0.2	0.005	78.63±28.51	100.15±3.32	6.24	3.53	14.34
Paclobutrazol	y = 19399416x + 35737	0.997±1.35	1.9	0.004	91.31±3.31	89.73±0.16	5.94	2.56	12.94
Pencycuron	y = 70947179x + 55253	0.999±1.57	2.0	0.004	91.50±5.73	92.07±0.37	1.70	6.01	12.49
Phenmedipham	y = 58432681x + 99091	0.997±1.18	-1.1	0.004	80.55±5.97	92.15±1.11	10.73	4.44	23.22
Phenthoate	y = 2525933x + 41110	0.999±0.78	15.1	0.003	65.10±13.97	91.82±2.52	6.36	1.11	12.91
Phosmet	y = 36507487x + 43041	0.998±0.49	1.2	0.004	85.31±4.61	90.82±5.24	5.69	9.51	22.16
Phosphamidon	y = 23178394x + 393	1.000±0.25	0.3	0.005	92.10±1.25	94.22±1.43	7.24	1.32	14.72
Phoxim	y = 26817651x + 34776	0.999±1.41	5.1	0.004	89.96±5.43	95.99±0.72	5.46	1.09	11.14
Picolinafen	y = 35156050x + 70164	0.999±0.20	-2.2	0.005	75.40±19.28	93.00±1.80	12.15	10.05	31.54
Picoxystrobin	y = 68285161x + 80499	0.998±0.69	-2.5	0.004	92.35±8.04	93.97±1.69	2.37	4.20	9.65
Pirimiphos-methyl	y = 35040944x + 18821	1.000±0.33	4.1	0.005	88.97±2.77	93.67±1.62	4.10	8.07	18.10
Prochloraz	y = 30756669x + 28592	0.999±0.05	-4.2	0.004	80.38±0.83	85.67±0.59	4.28	9.69	21.19
Profenofos	y = 14738591x + 24585	0.998±0.61	-4.3	0.004	83.31±15.53	93.64±0.37	12.76	1.79	25.77
Promecarb	y = 16884870x + 12782	0.999±0.96	-1.4	0.005	87.10±10.01	97.52±1.71	4.31	1.62	9.21
Prometon	y = 97154208x + 36497	1.000±0.79	2.8	0.005	88.16±1.24	93.30±1.05	2.94	3.00	8.40
Propetamophos	y = 6547422x + 12121	0.996±1.32	11.3	0.003	90.97±13.05	89.26±2.38	4.87	7.70	18.22
Propham	y = 3683967x + 2219	0.999±0.53	-1.3	0.005	96.09±4.92	102.87±2.34	4.64	7.24	17.20
Propiconazole	y = 3670108x + 10155	0.995±0.74	10.8	0.003	76.39±36.09	92.29±2.92	3.00	3.54	9.28
Propoxur	y = 50481801x + 9065	1.000±0.88	-0.6	0.005	96.96±4.04	98.81±1.32	6.60	4.10	15.54
Proquinazid	y = 33383152x + 94012	0.995±0.96	-5.2	0.004	68.24±35.87	73.99±2.08	4.21	8.00	18.08
Prosulfocarb	y = 104603585x + 82061	0.999±0.22	0.2	0.005	85.96±6.32	90.82±1.09	2.39	5.36	11.74
Pyracarbolid	y = 118588195x + 137355	0.999±0.12	3.6	0.004	83.84±2.83	90.95±0.96	4.35	3.65	11.36
Quinalphos	y = 9640112x + 7279	1.000±0.12	15.9	0.003	93.20±1.16	93.19±1.07	4.81	4.48	13.15
Quinmerac	y = 54774920x + 85245	0.999±0.67	-2.2	0.004	83.54±4.82	95.31±1.54	10.64	1.26	21.43
Quinoxyfen	y = 15728154x + 4347	1.000±1.13	-1.0	0.005	84.69±3.28	88.21±0.84	4.14	4.13	11.70
Rotenone	y = 2512408x + 4747	0.998±1.84	-3.6	0.004	92.55±7.16	95.54±2.33	6.21	8.70	21.38
Secbumeton	y = 82359990x + 42133	1.000±1.20	2.6	0.005	89.80±3.45	92.65±1.93	3.18	7.27	15.87
Silthiopham	y = 44620347x + 75384	0.998±1.96	15.8	0.004	65.46±5.72	77.69±1.19	3.14	1.69	7.13
Spirodiclofen	y = 8424717x + 15991	0.999±0.17	-4.3	0.005	79.58±28.65	78.29±4.36	5.11	2.70	11.56
Spiromesifen	y = 14604445x -720	0.999±0.02	-8.6	0.006	100.64±10.09	108.97±3.90	1.42	1.63	4.32
Spiroxamine	y = 85536698x + 45664	1.000±0.38	2.0	0.005	89.98±3.98	92.69±1.05	4.43	5.19	13.65
Sulfentrazone	y = 548398x + 535	1.000±0.32	10.5	0.005	71.78±19.19	81.65±0.98	8.40	6.58	21.34
Tebufenozid	y = 38191377x + 36318	0.999±0.62	-1.9	0.004	98.06±8.76	94.04±3.65	2.58	1.13	5.63
Tebuthiuron	y = 76864203x + 75882	0.999±0.83	1.2	0.004	87.19±3.89	93.85±0.87	5.70	3.27	13.14
Temephos	y = 9827292x + 5773	1.000±1.38	-1.8	0.005	86.89±16.83	92.11±1.60	7.16	2.55	15.20
Terbufos	y = 3198109x + 7047	0.997±1.77	13.0	0.004	69.26±16.66	81.16±0.80	8.54	10.07	26.41
Thiabendazol	y = 31270104x -48763	0.998±0.75	-9.2	0.006	85.65±2.23	92.97±2.97	5.40	1.05	11.00
Thidiazuron	y = 6308108x + 5184	0.999±1.27	8.4	0.004	77.11±9.24	85.80±2.25	3.11	4.42	10.81

Thiodicarb	y = 17000632x + 17394	0.999±1.07	2.6	0.004	65.13±3.69	73.07±1.25	0.86	1.27	3.07
Thiofanox	y = 1088129x + 1912	0.996±0.38	-4.1	0.005	77.03±7.75	91.45±4.37	8.07	2.30	16.78
Tolclofos-methyl	y = 2830930x + 3495	0.999±1.27	-0.6	0.005	77.72±15.41	96.20±2.67	9.93	8.01	25.52
Tralkoxydim	y = 15102766x + 31166	0.998±0.78	10.0	0.005	73.99±12.48	75.18±3.23	5.77	1.19	11.78
Triadimefon	y = 6907945x + 4322	0.999±0.30	-0.3	0.005	90.98±3.54	93.38±3.65	5.12	5.21	14.61
Triazophos	y = 92640755x + 99239	0.999±0.06	1.8	0.004	90.55±4.01	92.09±2.03	1.61	5.77	11.98
Trichlorfon	y = 744933x + -353	0.997±0.48	13.2	0.004	120.76±2.16	100.36±2.31	9.10	4.92	20.69
Tricyclazol	y = 74180503x + 15407	1.000±1.13	-5.7	0.005	91.13±3.05	94.48±1.50	8.50	4.83	19.55
Trietazin	y = 17062512x + 24124	0.998±0.95	2.3	0.004	85.65±3.59	94.22±1.20	5.19	3.39	12.40
Triflumizol	y = 57096448x + 62483	0.999±0.60	0.0	0.004	74.39±5.46	83.93±0.15	2.78	2.99	8.17
Triflumuron	y = 11141275x + 10926	1.000±1.25	1.3	0.004	93.61±5.10	95.52±3.29	8.21	2.25	17.03
Trimethacarb	y = 36381621x + 31863	0.999±0.72	-2.6	0.004	95.48±5.46	97.82±1.73	3.26	2.08	7.73
Triticonazole	y = 6466574x + 3064	0.999±0.68	0.6	0.004	100.16±13.47	93.48±1.55	9.20	2.57	19.10
Uniconazole-P	y = 16624897x + 41708	0.997±1.86	4.0	0.003	72.64±11.91	96.50±2.48	6.27	1.95	13.13
Vamidothion	y = 47935963x + 95094	0.996±1.78	11.6	0.004	75.11±6.30	77.50±40.79	5.14	5.71	15.37
