# Supplementary material for: Four-Year Monitoring Survey of Pesticide Residues in Tomato Samples: Human Health and Environmental Risk Assessment
Source: J Xenobiot. 2025 Oct 20;15(5):171. doi: 10.3390/jox15050171 (PMC12564937; doi:10.3390/jox15050171)
Supplement: Supplementary file 1 [file jox-15-00171-s001.zip › jox-3883729-supplementary/Table S2.pdf]

Table S2. Chronic risk assessment (JMPR methodology - IEDI/TMDI) for EU populations (Adult and General Population) using EFSA's PRIMo tool revision 3.1.

Pesticide		Population Group												
		G08*	ES adult	G10	G06	G07	PT general	G11	G15	DE women	DE general	FR adult	NL general	FI adult
acetamiprid	ADI	11%	8%	14%	36%	11%	9%	9%	12%	7%	7%	5%	4%	6%
	Exp	0.14	0.09	0.16	0.43	0.13	0.11	0.11	0.14	0.09	0.08	0.06	0.05	0.07
cymoxanil	ADI	0.4%	0.3%	0.5%	1%	0.4%	0.3%	0.3%	0.5%	0.3%	0.3%	0.2%	0.2%	0.2%
	Exp	0.06	0.04	0.07	0.18	0.05	0.04	0.05	0.06	0.04	0.03	0.02	0.02	0.03
metalaxyl	ADI	0.1%	0.1%	0.1%	0.3%	0.1%	0.1%	0.1%	0.1%	0.1%	0.0%	0.0%	0.0%	0.0%
	Exp	0.07	0.05	0.08	0.21	0.06	0.05	0.05	0.07	0.04	0.04	0.03	0.03	0.03
azoxystrobin	ADI	0.1%	0.0%	0.1%	0.2%	0.1%	0.1%	0.1%	0.1%	0.0%	0.0%	0.0%	0.0%	0.0%
	Exp	0.14	0.09	0.16	0.43	0.13	0.11	0.11	0.14	0.09	0.08	0.06	0.05	0.07
boscalid	ADI	1.0%	0.7%	1%	3%	0.9%	0.8%	0.8%	1%	0.6%	0.6%	0.4%	0.4%	0.5%
	Exp	0.39	0.27	0.47	1.22	0.37	0.30	0.31	0.41	0.25	0.22	0.16	0.14	0.19
mandipropamid	ADI	0.0%	0.0%	0.0%	0.1%	0.0%	0.0%	0.0%	0.0%	0.0%	0.0%	0.0%	0.0%	0.0%
	Exp	0.05	0.03	0.05	0.14	0.04	0.04	0.04	0.05	0.03	0.03	0.02	0.02	0.02
dimethomorph	ADI	0.2%	0.2%	0.3%	0.7%	0.2%	0.2%	0.2%	0.2%	0.1%	0.1%	0.1%	0.1%	0.1%
	Exp	0.11	0.08	0.14	0.36	0.11	0.09	0.09	0.12	0.07	0.07	0.05	0.04	0.06
myclobutanil	ADI	0.2%	0.1%	0.2%	0.6%	0.2%	0.1%	0.1%	0.2%	0.1%	0.1%	0.1%	0.1%	0.1%
	Exp	0.05	0.03	0.05	0.14	0.04	0.04	0.04	0.05	0.03	0.03	0.02	0.02	0.02
tetraconazole	ADI	2%	1%	2%	5%	2%	1%	1%	2%	1	1	0.7%	0.6%	0.8%
	Exp	0.07	0.05	0.08	0.21	0.06	0.05	0.05	0.07	0.04	0.04	0.03	0.03	0.03
penconazole	ADI	0.1%	0.1%	0.1%	0.4%	0.1%	0.1%	0.1%	0.1%	0.1%	0.1%	0.0%	0.0%	0.1%
	Exp	0.03	0.02	0.04	0.11	0.03	0.03	0.03	0.04	0.02	0.02	0.01	0.01	0.02
tebuconazole	ADI	0.5%	0.3%	0.5%	1%	0.4%	0.4%	0.4%	0.5%	0.3%	0.3%	0.2%	0.2%	0.2%
	Exp	0.14	0.09	0.16	0.43	0.13	0.11	0.11	0.14	0.09	0.08	0.06	0.05	0.07
zoxamide	ADI	0.0%	0.0%	0.0%	0.1%	0.0%	0.0%	0.0%	0.0%	0.0%	0.0%	0.0%	0.0%	0.0%
	Exp	0.13	0.09	0.15	0.39	0.12	0.10	0.10	0.13	0.08	0.07	0.05	0.05	0.06
Spinosad sum A+D	ADI	0.3%	0.2%	0.4%	1%	0.3%	0.3%	0.3%	0.3%	0.2%	0.2%	0.1%	0.1%	0.2%
	Exp	0.08	0.05	0.10	0.25	0.08	0.06	0.06	0.08	0.05	0.05	0.03	0.03	0.04
pyraclostrobin	ADI	0.2%	0.2%	0.3%	0.7%	0.2%	0.2%	0.2%	0.2%	0.1%	0.1%	0.1%	0.1%	0.1%
	Exp	0.07	0.05	0.08	0.21	0.06	0.05	0.05	0.07	0.04	0.04	0.03	0.03	0.03
clofentezin	ADI	0.2%	0.1%	0.2%	0.6%	0.2%	0.2%	0.2%	0.2%	0.1%	0.1%	0.1%	0.1%	0.1%
	Exp	0.03	0.02	0.04	0.11	0.03	0.03	0.03	0.04	0.02	0.02	0.01	0.01	0.02
difenoconazole	ADI	3.0%	2%	3.0%	8.0%	2.0%	2.0%	2.0%	3.0%	2.0%	2.0%	1%	1.0%	1.0%
	Exp	0.26	0.18	0.31	0.82	0.25	0.21	0.21	0.27	0.17	0.15	0.11	0.10	0.13
ametocratidin	ADI	0.0%	0.0%	0.0%	0.0%	0.0%	0.0%	0.0%	0.0%	0.0%	0.0%	0.0%	0.0%	0.0%
	Exp	0.13	0.09	0.15	0.39	0.12	0.10	0.10	0.13	0.08	0.07	0.05	0.05	0.06
metaflumizone	ADI	0.1%	0.1%	0.1%	0.4%	0.1%	0.1%	0.1%	0.1%	0.1%	0.1%	0.0%	0.0%	0.1%
	Exp	0.01	0.01	0.01	0.04	0.01	0.01	0.01	0.01	0.01	0.01	0.00	0.00	0.01
emamectin	ADI	5%	3%	5%	14%	4%	4%	4%	5%	3%	3%	2%	2%	2%
	Exp	0.02	0.02	0.03	0.07	0.02	0.02	0.02	0.02	0.01	0.01	0.01	0.01	0.01
etofenprox	ADI	0.2%	0.1%	0.2%	0.5%	0.1%	0.1%	0.1%	0.2%	0.1%	0.1%	0.1%	0.1%	0.1%
	Exp	0.05	0.03	0.05	0.14	0.04	0.04	0.04	0.05	0.03	0.03	0.02	0.02	0.02

*G08: General population (Austria. Germany. Poland and Spain). mean body weight 60 kg. ES adult: Adult (Spain) ≥17 years, mean body weight 77.1 kg. G10: General population (Bulgaria. Croatia. Cyprus. Estonia. Italy. Latvia and Malta),

mean body weight 60 kg. G06: General population (Greece), mean body weight 60 kg. PT general: General population (Portugal), mean body weight 60 kg. G11: General population (Belgium and the Netherlands), mean body weight 60 kg. G15: General population (Czech Republic. Denmark. Hungary. Ireland. Lithuania. Portugal. Romania. Slovakia. Slovenia and Sweden), mean body weight 60 kg. DE women: Women of childbearing (Germany) 14–50 years, mean body weight 67.5 kg. DE general: General population (Germany), mean body weight 76.4 kg. FR adult: Adults (France) ≥15 years, mean body weight 66.4 kg. NL general: General population (Netherlands), mean body weight 65.8 kg. FI adult: Adults (Finland), mean body weight 77.1 kg.
