# Supplementary material for: Four-Year Monitoring Survey of Pesticide Residues in Tomato Samples: Human Health and Environmental Risk Assessment
Source: J Xenobiot. 2025 Oct 20;15(5):171. doi: 10.3390/jox15050171 (PMC12564937; doi:10.3390/jox15050171)
Supplement: Supplementary file 1 [file jox-15-00171-s001.zip › jox-3883729-supplementary/Table S3.pdf]

Table S3. Chronic risk assessment for EU populations (JMPR methodology - IEDI/TMDI) (Children, toddler and infant) using EFSA's PRIMo tool revision 3.1.

Pesticide		Population Group								
		ES* child	FR child	DE child	NL toddler	FR toddler	NL child	FI child 3 yr	FR infant	FI child 6 yr
acetamiprid	ADI	10%	8%	10%	10%	5%	6%	6%	0.9%	4%
	Exp	0.12	0.10	0.12	0.12	0.06	0.07	0.07	0.01	0.05
cymoxanil	ADI	0.4%	0.3%	0.4%	0.4%	0.2%	0.2%	0.2%	0.0%	0.2%
	Exp	0.05	0.04	0.05	0.05	0.02	0.03	0.03	0.00	0.02
metalaxyl	ADI	0.1%	0.1%	0.1%	0.1%	0.0%	0.0%	0.0%	0.0%	0.0%
	Exp	0.06	0.05	0.06	0.06	0.03	0.03	0.03	0.01	0.03
azoxystrobin	ADI	0.1%	0.1%	0.1%	0.1%	0.0%	0.0%	0.0%	0.0%	0.0%
	Exp	0.12	0.10	0.12	0.12	0.06	0.07	0.07	0.01	0.05
boscalid	ADI	0.8%	0.7%	0.8%	0.8%	0.4%	0.5%	0.5%	0.1%	0.4%
	Exp	0.33	0.28	0.34	0.34	0.16	0.19	0.19	0.03	0.15
mandipropamid	ADI	0.0%	0.0%	0.0%	0.0%	0.0%	0.0%	0.0%	0.0%	0.0%
	Exp	0.04	0.03	0.04	0.04	0.02	0.02	0.02	0.00	0.02
dimethomorph	ADI	0.2%	0.2%	0.2%	0.2%	0.1%	0.1%	0.1%	0.0%	0.1%
	Exp	0.10	0.08	0.10	0.10	0.05	0.06	0.06	0.01	0.04
myclobutanil	ADI	0.2%	0.1%	0.2%	0.2%	0.1%	0.1%	0.1%	0.0%	0.1%
	Exp	0.04	0.03	0.04	0.04	0.02	0.02	0.02	0.00	0.02
tetraconazole	ADI	1%	1%	1%	1%	0.7%	0.8%	0.8%	0.1%	0.7%
	Exp	0.06	0.05	0.06	0.06	0.03	0.03	0.03	0.01	0.03
penconazole	ADI	0.1%	0.1%	0.1%	0.1%	0.0%	0.1%	0.1%	0.0%	0.0%
	Exp	0.03	0.03	0.03	0.03	0.01	0.02	0.02	0.00	0.01
tebuconazole	ADI	0.4%	0.3%	0.4%	0.4%	0.2%	0.2%	0.2%	0.0%	0.2%
	Exp	0.12	0.10	0.12	0.12	0.06	0.07	0.07	0.01	0.05
zoxamide	ADI	0.0%	0.0%	0.0%	0.0%	0.0%	0.0%	0.0%	0.0%	0.0%
	Exp	0.11	0.09	0.11	0.11	0.05	0.06	0.06	0.01	0.05
Spinosad sum A+D	ADI	0.3%	0.2%	0.3%	0.3%	0.1%	0.2%	0.2%	0.0%	0.1%
	Exp	0.07	0.06	0.07	0.07	0.03	0.04	0.04	0.01	0.03
pyraclostrobin	ADI	0.2%	0.2%	0.2%	0.2%	0.1%	0.1%	0.1%	0.0%	0.1%
	Exp	0.06	0.05	0.06	0.06	0.03	0.03	0.03	0.01	0.03
clofentezin	ADI	0.2%	0.1%	0.2%	0.2%	0.1%	0.1%	0.1%	0.0%	0.1%
	Exp	0.03	0.03	0.03	0.03	0.01	0.02	0.02	0.00	0.01
difenoconazole	ADI	2%	2%	2%	2%	1%	1%	1%	0.2%	1%
	Exp	0.23	0.19	0.23	0.23	0.11	0.13	0.13	0.02	0.10
ametocratidin	ADI	0.0%	0.0%	0.0%	0.0%	0.0%	0.0%	0.0%	0.0%	0.0%
	Exp	0.11	0.09	0.11	0.11	0.05	0.06	0.06	0.01	0.05
metaflumizone	ADI	0.1%	0.1%	0.1%	0.1%	0.0%	0.1%	0.1%	0.0%	0.0%
	Exp	0.01	0.01	0.01	0.01	0.00	0.01	0.01	0.00	0.00
emamectin	ADI	4%	3%	4%	4%	2%	2%	2%	0.4%	2%
	Exp	0.02	0.02	0.02	0.02	0.01	0.01	0.01	0.00	0.01
etofenprox	ADI	0.1%	0.1%	0.1%	0.1%	0.1%	0.1%	0.1%	0.0%	0.1%
	Exp	0.04	0.03	0.04	0.04	0.02	0.02	0.02	0.00	0.02

*ES child: Children (Spain) 7–12 years. mean body weight 34.5 kg. FR child: Children (France) 3–15 years. mean body weight 18.9 kg. DE child: Children (Germany) 2–5 years. mean body weight 16.2 kg. NL toddler: Toddler (Netherlands) 8–

20 months. mean body weight 10.2 kg. FR toddler: Toddler (France) 25–36 months. mean body weight 13.6 kg. NL child: Children (Netherlands) 2–6 years. mean body weight 18.4 kg. FI child 3: Children (Finland) 3 years. mean body weight 15.2 kg. FR infant: Infant (France) 7–18 months. mean body weight 9.1 kg. FI child 6: Children (Finland) 6 years. mean body weight 22.4 kg.
