# Supplementary material for: Four-Year Monitoring Survey of Pesticide Residues in Tomato Samples: Human Health and Environmental Risk Assessment
Source: J Xenobiot. 2025 Oct 20;15(5):171. doi: 10.3390/jox15050171 (PMC12564937; doi:10.3390/jox15050171)
Supplement: Supplementary file 1 [file jox-15-00171-s001.zip › jox-3883729-supplementary/Table S4.pdf]

Table S4. Consumer acute risk assessment based on the HQ approach.

		**Adult**		**Toddler**	
Pesticide	ARfD (mg/kg/d)	EDI (mg/kg bw/d)	HQ	EDI (mg/kg bw/d)	HQ
Acetamiprid	0.005*	1.26E-04	2.53E-02	1,25E-04	2.49E-02
Acetamiprid	0.025	1.26E-04	5.05E-03	1,25E-04	4.98E-03
Cymoxanil	0.08	5.39E-05	6.74E-04	5,32E-05	6.65E-04
Metalaxyl	0.5	6.48E-05	1.30E-04	6,39E-05	1.28E-04
Azoxystrobin	NN	1.18E-04		1,17E-04	
Boscalid	3	3.49E-04	1.16E-04	3,44E-04	1.15E-04
Mandipropamid	NN	3.77E-05		3,71E-05	
Dimethomorph	0.6	1.02E-04	1.70E-04	1,01E-04	1.68E-04
Myclobutanil	0.31	4.06E-05	1.31E-04	4,00E-05	1.29E-04
Tetraconazole	0.05	5.58E-05	1.12E-03	5,50E-05	1.10E-03
Penconazole	0.5	2.49E-02	5.62E-05	2,77E-05	5.54E-05
Tebuconazole	0.03	4.09E-03	3.94E-03	1,17E-04	3.89E-03
Zoxamide	NN	7.99E-04		1,06E-04	
Spinosad (sum A + D)	0.1	5.84E-04	7.44E-04	7,34E-05	7.34E-04
Pyraclostrobin	0.03	8.61E-03	2.11E-03	6,25E-05	2.08E-03
Clofentezin	NN	2.48E-04		3,35E-05	
Difenoconazole	0.16	2.02E-03	1.46E-03	2,30E-04	1.44E-03
Ametoctradin	NN	1.60E-03		1,06E-04	
Metaflumizone	0.13	1.38E-02	7.80E-05	1,00E-05	7.69E-05
Emamectin	0.01	2.49E-02	2.26E-03	2,23E-05	2.23E-03
Etofenprox	1	4.04E-05	4.04E-05	3,98E-05	3.98E-05

* ADI changed in 2024; NN not necessary EFSA conclusions.
