# Supplementary material for: Four-Year Monitoring Survey of Pesticide Residues in Tomato Samples: Human Health and Environmental Risk Assessment
Source: J Xenobiot. 2025 Oct 20;15(5):171. doi: 10.3390/jox15050171 (PMC12564937; doi:10.3390/jox15050171)
Supplement: Supplementary file 1 [file jox-15-00171-s001.zip › jox-3883729-supplementary/Table S5.pdf]

Table S5. Acute risk assessment for EU populations (toddler and adults) using EFSA's PRIMo tool revision 3.1.

				Toddler		adults	
**Pesticides**	**ADI**	**ARfD**	**HR**	**IESTI**	**ARfD(%)**	**IESTI**	**ARfD(%)**
	**(mg/kg bw/d)**		**(mg/ kg)**	**(mg/kg bw/day)**		**(mg/kg bw)**	
Acetamiprid*	0.005	0.005	0.24	0.0063	**125.4%**	0.0023	45.9%
Acetamiprid	0.025	0.025	0.24	0.0140	55.8%	0.0038	15.2%
Cymoxanil	0.013	0.08	0.06	0.0016	2.0%	0.0006	0.7%
Metalaxyl	0.08	0.5	0.14	0.0037	0.7%	0.0013	0.3%
Azoxystrobin	0.2	NN*	0.23				
Boscalid	0.04	3	0.61	0.0159	0.5%	0.0058	0.2%
Mandipropamid	0.15	NN	0.07				
Dimethomorph	0.05	0.6	0.19	0.0050	0.8%	0.0018	0.3%
Myclobutanil	0.025	0.31	0.04	0.0010	0.3%	0.0004	0.1%
Tetraconazole	0.004	0.05	0.08	0.0021	4.2%	0.0008	1.5%
Penconazole	0.03	0.5	0.03	0.0008	0.2%	0.0003	0.1%
Tebuconazole	0.03	0.03	0.60	0.0157	52.2%	0.0057	19.1%
Zoxamide	0.5	NN	0.25				
Spinosad	0.024	0.1	0.31	0.0081	8.1%	0.0030	3.0%
Pyraclostrobin	0.03	0.03	0.43	0.0112	37.4%	0.0041	13.7%
Clofentezin	0.017	NN	0.05	0.0013	7.7%	0.0005	2.8%
Difenoconazole	0.01	0.16	0.23	0.0060	3.8%	0.0022	1.4%
Ametocratidin	10	NN	0.41				
Metaflumizone	0.01	0.13	0.01	0.0003	0.2%	0.0001	0.1%
Emamectin	0.0005	0.01	0.03	0.0008	7.8%	0.0003	2.9%
Etofenprox	0.03	1	0.07	0.0018	0.2%	0.0007	0.1%

*Not necessary EFSA conclusions.
