# Supplementary material for: Four-Year Monitoring Survey of Pesticide Residues in Tomato Samples: Human Health and Environmental Risk Assessment
Source: J Xenobiot. 2025 Oct 20;15(5):171. doi: 10.3390/jox15050171 (PMC12564937; doi:10.3390/jox15050171)
Supplement: Supplementary file 1 [file jox-15-00171-s001.zip › jox-3883729-supplementary/Table S6.pdf]

Table S6. Table of all available data on the acute toxicity to aquatic invertebrates.

Pesticides	AF	EC_{50} *Daphnia Magna 48-hour* (mg/L)	reference
Acetamiprid	10	49.80	EFSA Journal 2016;14(11):4610 [43]
Cymoxanil	10	27.00	EFSA Scientific Report (2008) 167 [44]
Metalaxyl	10	28.00	U.S. Environmental Protection Agency. https://archive.epa.gov/pesticides/chemicalsearch/chemical/foia/web/pdf/113501/113501-068.pdf [45]
Azoxystrobin	10	0.11	EFSA Journal 2010; 8(4):1542 [46]
Boscalid	10	5.33	Australian Pesticides and Veterinary Medicines Authority 2004 [47]
Mandipropamid	10	7.10	EFSA Journal 2012;10(11):2935 [48]
Dimethomorph	10	10.60	EFSA Scientific Report (2006) 82[49]
Myclobutanil	10	17.00	EFSA Journal 2010;8(10):1682 [50]
Tetraconazole	10	3.00	EFSA Scientific Report (2008) 152 [51]
Penconazole	10	6.75	EFSA Scientific Report (2008) 175 [52]
Tebuconazole	10	2.79	EFSA Scientific Report (2008) 176 [53]
Zoxamide	10	0.69	EFSA Journal 2017;15(9):4980 [54]
Spinosad (Sum A + D)	10	7.30	EFSA Journal 2018;16(4):5252 [55]
Pyraclostrobin	10	0.02	EFSA Journal. 2025;23:e9257 [56]
Clofentezin	10	71.63	EFSA Journal 2021;19(8):6817 [57]
Difenoconazole	10	0.77	EFSA Journal 2011;9(1):1967 [58]
Ametoctradin	10	0.59	EFSA Journal 2012;10(11):2921 [59]
Metaflumizone	10	0.33	EFSA Journal 2013;11(10):3373 [60]
Emamectin	10	0.001	EFSA Journal 2012;10(11):2955 [61]
Etofenprox	10	0.0012	EFSA Scientific Report (2008) 213 [62]
