# Supplementary figures and images for: Four-Year Monitoring Survey of Pesticide Residues in Tomato Samples: Human Health and Environmental Risk Assessment
Source: J Xenobiot. 2025 Oct 20;15(5):171. doi: 10.3390/jox15050171 (PMC12564937; doi:10.3390/jox15050171)

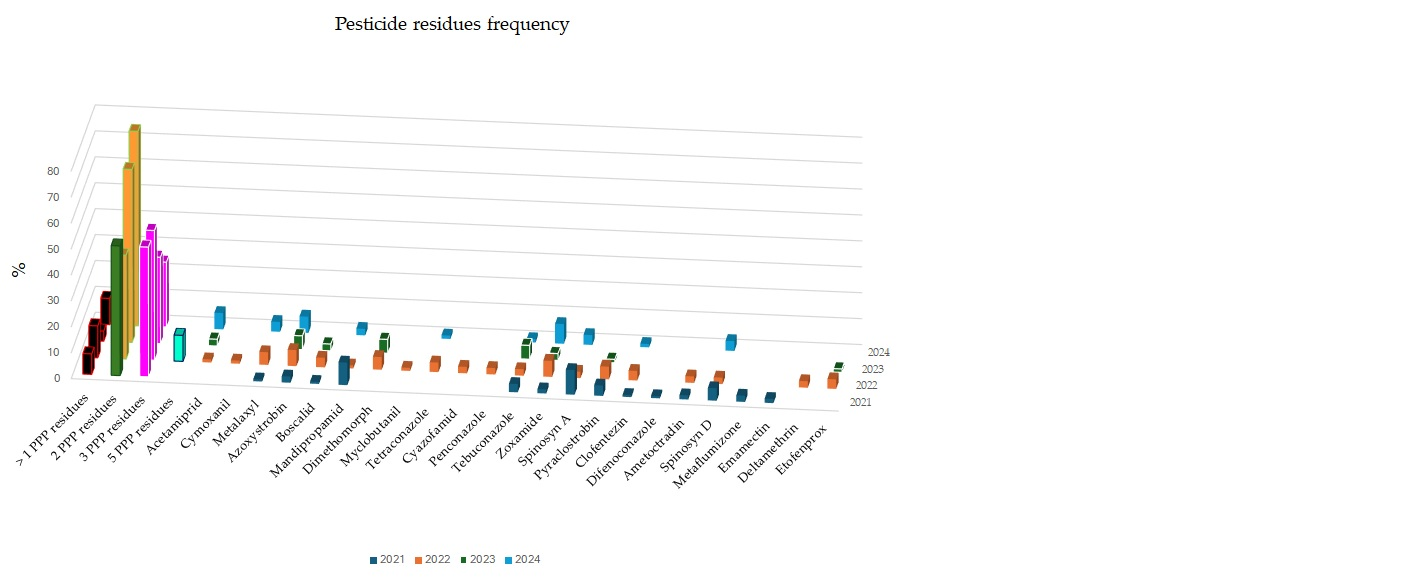

Supplement: Supplementary file 1 [file jox-15-00171-s001.zip › jox-3883729-supplementary/Figure S1_Pesticide Frequency.png]
